# Clinical characteristics and proposed mechanism of pediatric spinal cord injury resulting from backbend practice

**DOI:** 10.3389/fped.2023.1263280

**Published:** 2023-10-10

**Authors:** Shuang Guo, Huiming Gong, Peipei Xu, Yang Xie, Degang Yang, Zitong Liu, Yuwei Yang, Liang Chen, Yongqi Xie, Mingliang Yang

**Affiliations:** ^1^ School of Rehabilitation, Capital Medical University, Beijing, China; ^2^ China Rehabilitation Research Center, Beijing, China; ^3^ Rehabilitation Department, Ruijin Hospital, Shanghai Jiaotong University School of Medicine, Shanghai, China; ^4^ Beijing Opera Art’s College, Beijing, China; ^5^ Center of Neural Injury and Repair, Beijing Institute for Brain Disorders, Beijing, China

**Keywords:** spinal cord injury, children, backbend, dance, femoral nerves

## Abstract

**Objective:**

Pediatric spinal cord injury without radiographic abnormality (SCIWORA) caused by backbend practice is increasing. This study proposed an underlying ‘combined injury mechanism’ related to the spinal cord and femoral nerve overstretching.

**Methods:**

Pediatric patients diagnosed with backbend-associated SCIWORA at the China Rehabilitation Research Center during 2017–2021 were recruited. Clinical and imaging data were collected, and each patient's clinical course and prognosis were determined. Healthy dancers were recruited to simulate the backbend, obtain images, and estimate the spinal cord and femoral nerve stretch ratio. A model for the ‘combined injury mechanism’ was established using 4-week-old SD rats.

**Results:**

Forty-two SCIWORA female patients with an average age of 6 (SD 1) years and an average hospitalization time of 91 (SD 43) days were assessed. The primary initial symptom was pain in the back and/or lower extremities (33, 79%). The average time from injury onset to severe paralysis was 2.0 (SD 0.6) hours. Most patients had complete paraplegia (32, 76%), and neurological levels were distributed mainly in thoracic segments (38, 91%). Patients with elicited tendon reflexes on admission tended to have an incomplete spinal cord injury (*p* = 0.001) and improved motor recovery (*p* = 0.018). After one year, the most common complications were scoliosis (31, 74%) and abnormal hips (14, 33%). Injury of the caudal spinal cord torn by nerve roots was confirmed by surgical exploration in a case. The thoracic spinal cord and femoral nerves were overstretched by 148.8 ± 3.6% and111.7 ± 4.0%, respectively, in a full backbend posture. The ‘combined injury mechanism’ was partially replicated in the animal model.

**Conclusion:**

Spinal overstretch and transient dislocation are considered the primary mechanisms by which SCIWORA occurs in children. Overstretching the femoral nerve aggravates spinal cord injuries caused by backbend practice.

## Introduction

Backbend involves bending the spine backward from standing until the hands touch the ground, maintaining the body arch, and then returning to the upright position. It is a very common physical practice in dance and yoga practice (1). However, this action can cause severe spinal cord injury without radiographic abnormality (SCIWORA), particularly among female children (2). In China, the incidence of pediatric SCIWORA due to dance has increased by 6.2% in sports injury in recent years (3–6).

The initial symptom of SCIWORA is back pain, which typically occurs during the backbend and is immediately followed by radiating pain, numbness, and weakness in the lower extremities (2). This can progress to severe flaccid paraplegia within 2–3 h. Early contrast-enhanced magnetic resonance imaging (MRI) examination shows only a slight hyperintensity (edema) in the thoracolumbar spinal cord (7). Given the absence of obvious trauma and the progressive aggravated neurological symptoms, children are often misdiagnosed as having myelitis, vasogenic ischemic injury, or other disorders.

The current mechanism for backbend-associated SCIWORA is thought to involve an excessive longitudinal stretching of the spinal cord and/or transient spinal dislocation that resembles pediatric SCIWORA associated with violent events such as traffic accidents or falls (8). However, most patients have undergone long, repeated backbend training, have no obvious trauma (real-time video recording, see additional information 1), and experience radiating pain in the anterior thigh and medial leg which are innervated by femoral nerves (the second to fourth lumbar nerve roots, L2–4). Thus, we speculated that backbend-associated SCIWORA might occur through a ‘combined injury mechanism’ involving three aspects: (a) overstretching of the spinal cord and femoral nerve while maintaining a “backbend” posture, (b) transient spinal dislocation, and (c) muscle dyssynergia during which, for example, overstretching of the femoral nerves is exacerbated by a sudden eccentric contraction of the iliopsoas and quadriceps.

To confirm this proposed mechanism, we investigated a group of clinical cases and recruited volunteer dancers to imitate the full backbend and measure changes in the lengths of the spine, spinal cord, and femoral nerve. We then replicated this mechanism for femoral nerve distraction injury using an animal model. The findings of this study will enhance current knowledge about SCIWORA related to backbend practice and help to inform prevention strategies.

## Materials and methods

### Clinical study design

In this observational study (Clinical Registration No: ChiCTR2100051211), clinical cases admitted to the China Rehabilitation Research Center were obtained prospectively from Feb 2017 to Dec 2021. The scheduled follow-up period was one-year post-injury. The study was approved by the China Rehabilitation Research Center Ethics Committee (No: 2018-005-1) and written informed consent was obtained from legal representatives of eligible patients prior to enrolment.

Inclusion criteria included (a) initial neurological symptoms occurring ≤2 h after backbend practice, (b) no history of violent injury ≤2 weeks before the onset of neurological symptoms, (c) spine X-ray, CT, and MRI results showing a normal spine that lacked deformities, bony structures, intervertebral discs, and ligament damage, and (d) at least one MRI examination 3 months after the injury.

Evaluation data included (a) demographic information (gender, age, and dance age), (b) case history (any discomfort 2 weeks before the injury, posture control lost during the backbend, location of the initial symptoms, and incubation period from injury to paralysis onset), (c) neurological characteristics (level of nerve lesion, neurological impairment scale [American Spinal Injury Association Impairment Scale(AIS) (9)], muscle strength and tension[Ashworth scale (10), tendon reflex, anal reflex, and 4] imaging information. Dance age refers to how long a patient has been practicing dancing at the time of injury.

## Mechanism research

### Simulation of the backbend movement in volunteer dancers

**Figure 1:**
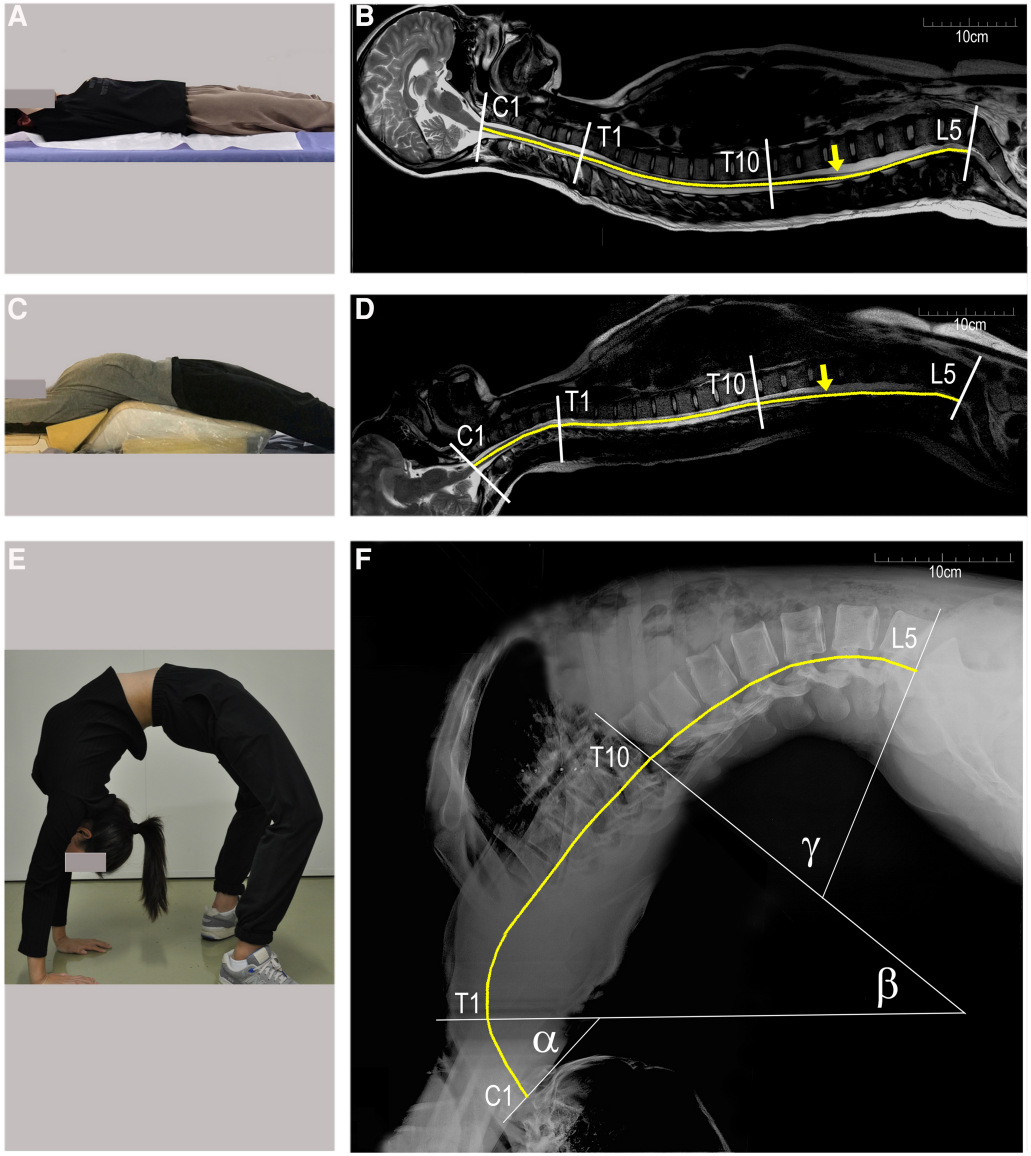
Changes in the spinal length of a healthy female dancer (16 years of age, 168 cm in height). (**A-D**) sagittal MRI of the whole spine in the supine and partial backbend positions; (**E** and **F**) lateral x-rays of a full backbend. The spine is divided into three segments: C1–C7 segments with angle α, T1–T10 with angle β, and T11–L5 with angle γ (**F**) the end of the conus medullaris is marked by yellow arrows in (**B** and **D**). The length of the spinal cord is estimated by the curved yellow lines. C1, first cervical vertebra; T1, first thoracic vertebra; L5, fifth lumbar vertebra.

Healthy volunteer dancers who have normal spine and spinal cord (aged 15.7 ± 1.5 years old, 162.3 ± 5.1 cm in height, weighing 52 ± 5.3 kg in weight) and having undergone 19 ± 4.6 months of dance training were recruited to simulate the backbend movement so that changes in the spine, spinal cord, and femoral nerve length could be observed. Lateral x-rays of the whole spine were obtained in the supine position (lying flat) and during a full backbend (Sagittal Cobb Angle (C1-L5), 151 ± 3.6°) (Figures 1A,E), and the entire spine MRI were performed as the volunteers were lying flat and in a partial backbend posture (56.0 ± 12.4°) (Figures 1A,C). The length of the femoral nerve was measured from the second lumbar disc to the tibial tubercle according to its anatomy using RadiAnt DICOM Viewer software (Poland).

### Simulation of femoral nerve traction injury in animal model

**Figure 2:**
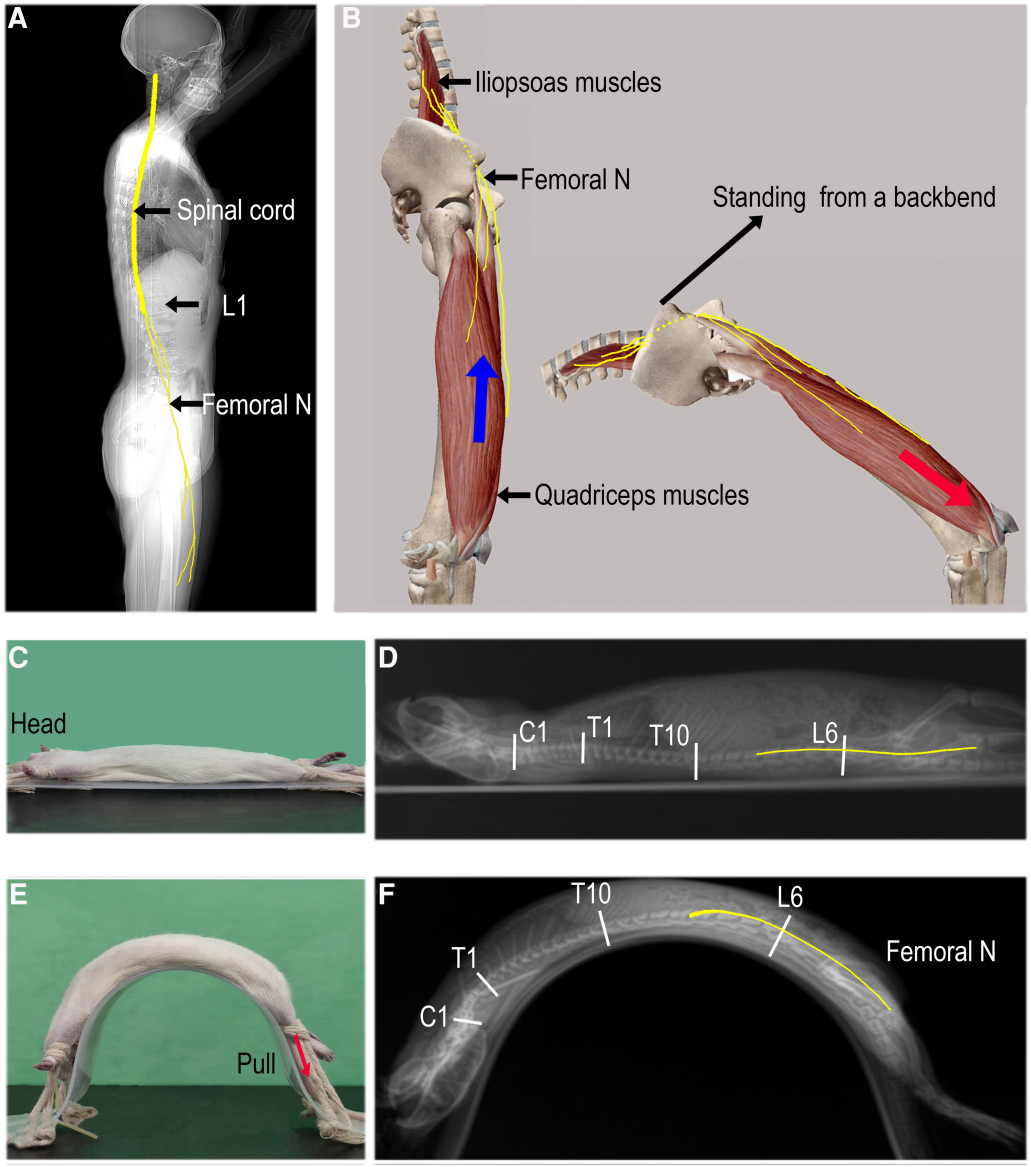
Mechanism of femoral nerve overstretching. (**A**) The spinal cord (C1–L1) and femoral nerves (originating from L2–L4 nerve roots). (**B**) The femoral nerves are overstretched from a standing position to a full backbend and are further pulled distally if a sudden eccentric contraction of the quadriceps occurs (red arrow). (**C** and **E**) In the supine and passive full backbend positions, respectively. The unilateral low limb is rapidly pulled distally to stretch the femoral nerves (red arrow); (**D** and **F**) a lateral x-ray of the whole spine with estimated lengths of the spine and femoral nerves (yellow lines).

Four-week-old SD rats (117.5 ± 5.0 g) equivalent in age to human children were used as the animal model. After receiving inhalation anesthesia (2% isoflurane), lateral spine x-rays were taken in the supine and full backbend position (fixed on a special arch plate; Figures 2C,E). Rats were placed in both postures and one unilateral lower extremity was suddenly stretched by applying a pulling force to the rat's ankle to simulate femoral nerve traction injury under a sudden eccentric contraction of the iliopsoas and quadriceps muscles. The extra force, up to 25N, was repeated 3–4 times during a period of 10 s. The animal experiment protocol was approved by the Experimental Animal Centre of the Capital Medical University of China (Approval No. AEEI-2022-085) and conducted according to the ethical rules of the Animal Experiments and Experimental Animal Welfare Committee. All procedures in this study were performed in compliance with ARRIVE guidelines (11). RadiAnt DICOM Viewer software (Poland) was used to measure the length.

### Statistical analysis

SPSS (version 24.0) was used for the statistical analysis. Continuous measurements are presented as mean (SD) if they were normally distributed or median (IQR) if they were not, and categorical variables are presented as counts (%). When the paired samples conformed to the normal distribution, paired samples *t*-tests were used, otherwise, the nonparametric rank-sum test was used. A chi-square test was used for categorical data. *P* < 0.05 was considered statistically significant.

## Results

### Clinical aspects

A total of 42 girls with SCIWORA resulting from backbend practice were included in this study, all of whom were 6 ± 1 year of age. Their dance age was 17 ± 14 months, of whom 36% were >1 year, and most were skilled at the backbend. Initial symptoms included numbness in the lower extremities (64%), immediate back pain (24%), abdominal pain (7%), and radiating pain along the anterior thigh and/or medial leg. These symptoms appeared while doing or immediately following a backbend. Lower extremity weakness manifested as progressive aggravation and mostly resulted in severe paralysis within 24 h (2.0 ± 0.6 h). Most patients (69%) recalled losing control of the backbend posture after sudden pain or discomfort in the back (Table 1).

**Table 1:** Clinical characteristics.

Criteria	Patients (*n* = 42)
Sex	
Female	42
Male	0
Age (years)	
Mean (SD)	6 ± 1
Range	4–8
Rehabilitation hospitalization time (days)	
Mean (SD)	91 ± 43
Range	2–218
Dance age (months)	
Mean (SD)	17 ± 14
Range	1–60
<12	8 (19%)
≥12	15 (36%)
Not clear	19 (45%)
Discomfort (within two weeks before the injury)	
Normal	32 (76%)
Cough	4 (9%)
Fever	2 (5%)
Pharyngalgia	2 (5%)
Others	2 (5%)
Lost posture control when doing a backbend	
Yes	29 (69%)
No	13 (31%)
Latent period before paralysis (hours)	
Mean (SD)	2.0 ± 0.6
Range	0–24
Immediate	9 (21%)
<0.5	7 (17%)
0.5–1	9 (21%)
>1	17 (41%)
Location of initial symptoms	
Lower extremity	22 (52%)
Back	6 (14%)
Abdominal	2 (5%)
Back & lower extremity	4 (10%)
Abdominal & lower extremity	1 (2%)
Others	3 (7%)
Normal	4 (10%)
Initial diagnosis	
Spinal cord injury	9 (22%)
Acute myelitis	8 (19%)
SCIWORA^a^	6 (14%)
Tumor	1 (2%)
Others	5 (12%)
Not clear	13 (31%)
Complications (one year later)^b^	
Urinary tract infection	8 (19%)
Scoliosis	31 (74%)
Abnormal hips^c^	14 (33%)
Bedsore, pneumonia, neuralgia, etc.	11 (26%)

^a^ SCIWORA, spinal cord injury without radiographic abnormality.

^b^ 22 patients had ≥1 complication.

^c^ Abnormal hips include hip dysplasia, hip subluxation and hip dislocation.

Two-thirds of the patients (76%) suffered a complete spinal cord injury (ASIA, grade A) on admission that was characteristic of flaccid paraplegia. Distribution of the neurological level within the lower thoracic cord (T7–12) and the upper thoracic cord (T1–6) occurred in 62% and 29% of cases, respectively (Table 2). Nineteen percent of the patients were initially misdiagnosed with acute myelitis (Table 1).

**Table 2:** Neurological examination.

Criteria	Admission	Discharge	*P*-value
Neurological level			
T1–T6	12 (29%)	11 (26%)	1
T7–T12	26 (62%)	28 (67%)	
L1–S5	4 (9%)	3 (7%)	
Neurological impairment scale (AIS^a^)			
A	32 (76%)	32 (76%)	0.034
B	3 (7%)	1 (3%)	
C	5 (12%)	3 (7%)	
D	2 (5%)	6 (14%)	
The motor score of lower extremities			
Median (IQR)	0 (0–0)	0 (0–0)	0.008
Range	0–32	0–40	
The muscle tone of lower extremities (Ashworth scale)			
Low	30 (71%)	30 (71%)	0.655
Normal	9 (22%)	9 (22%)	
1	3 (7%)	2 (5%)	
2	0 (0)	1 (2%)	
Anal reflex			
Absent	35 (83%)	34 (81%)	0.317
Elicited	7 (17%)	8 (19%)	
Tendon reflex^b^			
Absent	32 (76%)	32 (76%)	1
Elicited	10 (24%)	10 (24%)	

^a^ AIS, American Spinal Injury Association Impairment Scale.

^b^ The distribution of patients on admission according to the neurological impairment scale, *P* = 0.001.

### Imaging information

**Figure 3:**
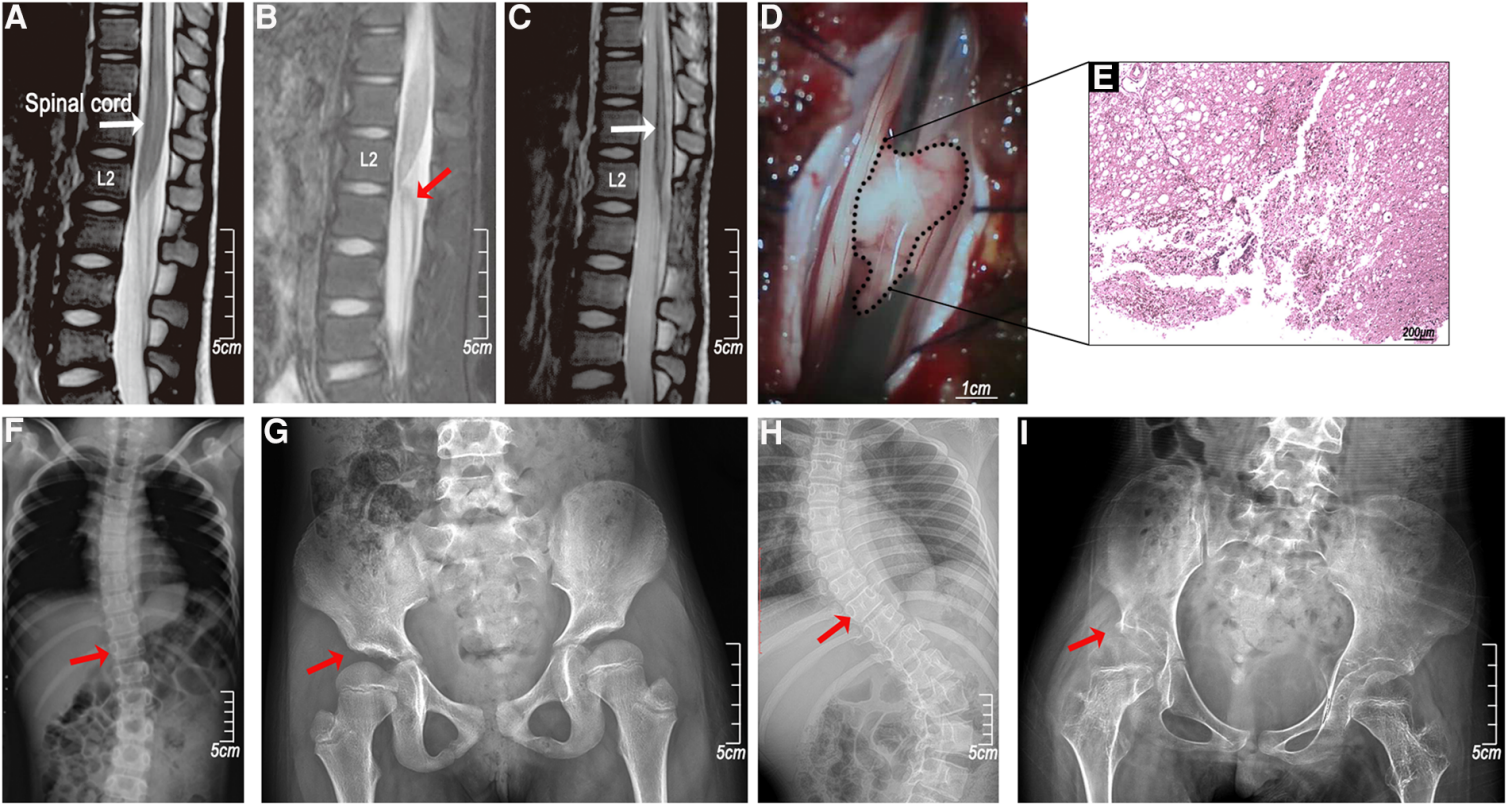
A clinical case of femoral nerve overstretching in a 5-year-old female child. (**A**) An MRI (T2 sequences) at 17 h showed no significant abnormalities in the spinal cord; (**B**) the MRI (T2 sequences) at 85 h showed a protrusion near the conus medullaris; (**C**) the MRI (T2 sequences) at 6 weeks after the injury showed spinal cord atrophy; (**D**) surgical exploration showed a conus medullaris tear injury; (**E**) HE staining of the protrusion (necrotic tissue infiltrated by inflammatory cells); (**F** and **H**) right hip dysplasia (red arrow) and (**G** and **I**) thoracic scoliosis (red arrow) at years 1 and 5.

X-ray and MRI examinations of the whole spine showed that the bone, intervertebral disc, and ligament structures were normal. There were no spinal cord deformities, tethered spinal cord, low-lying conus medullaris, or vascular malformation in these cases. In the early MRI (within one week) of the spinal cord (T2 sequences), multisegmented, uneven distribution, and mildly enhanced abnormal signals were present in the thoracolumbar spinal cord, and in some cases, slight swelling in the conus medullaris with disordered cauda equina. In the late MRI (over 3 months), nearly all the cases had severe spinal cord atrophy. In a specifical case, the MRI showed no significant abnormities at 17 h post-injury (Figure 3A) and revealed a space-occupying lesion just below the conus medullaris by 85 h (Figure 3B). Subsequent surgical exploration confirmed that the lumbar-sacral cord was torn by the nerve roots and the “occupying lesion” was necrotic as a result of the injury (Figure 3D). Severe atrophy of the thoracolumbar spinal cord appeared 6 weeks later (Figure 3C) and scoliosis and hip dysplasia occurred after a year and five years (Figures 3F–I).

### Prognosis and complications

All patients were followed for at least one year. The median time of follow-up was 4(IQR 2.8–5.4) years. The follow-up at discharge (about six months post-injury) and one-year post-injury revealed that the prognosis was poor. Two-thirds of cases (76%) still had complete flaccid paraplegia with the decline of only a few sensory levels. Cases with tendon reflexes (knee and Achilles tendon) most often had incomplete spinal cord injury (*p* = 0.001) on admission, and the recovery of motion function at discharge was improved over patients with absent tendon reflexes (*p* = 0.018) (Tables 2, 3). Early complication was mainly urinary tract infections (8, 19%). Late common complications (one year later) included abnormal hip (hip dysplasia, hip subluxation, and hip dislocation) (14, 33%) and scoliosis (31, 74%) (Table 1).

**Table 3:** Correlation between tendon reflex and prognosis (10 matched patients).

Patient	Tendon reflex	Age (years)	Hospitalization (days)	Neurological impairment scale				Motor scores in lower extremities		
				AIS A	AIS B	AIS C	AIS D	Admission	Discharge	d
10	Elicited	6 ± 1	102 ± 39	3	2	3	2	0 (0–15)	24 (0–40)	9 (0–31)
10	Absent	6 ± 1	105 ± 32	7	1	2	0	0 (0–0)	0 (0–0)	0 (0–0)
	*P*-value	0.083	0.55	0.22				0.068	0.017	0.018

### Changes in spine, spinal cord and femoral nerve lengths caused by backbend

There was little change in the longitudinal length of the spine and spinal cord as a result of moving from lying flat to a partial backbend (Figures 1B,D, Table 4). When reaching a full-backbend posture, the total spine of the healthy volunteers increased to 126 ± 0.8% (69.1 ± 1.3 cm/54.8 ± 1.2 cm), during which the cervical (C1–7), thoracic (T1–10), and thoracolumbar segments (T11–L5) were 110.5 ± 2.0% (12.6 ± 0.8 cm/11.4 ± 0.5 cm), 148.8 ± 3.6% (30.4 ± 3.1 cm/20.5 ± 2.2 cm), and 113.6 ± 3.2% (26.0 ± 1.7 cm/22.9 ± 1.3 cm), respectively (Figures 1B,F, Table 4). The thoracic segment showed the most obvious change in length. The length of the femoral nerve, from the second lumbar disc to the tibial tubercle, was estimated to increase by111.7 ± 4.0% (69.4 ± 8.9 cm/61.9 ± 6.0 cm) during a full backbend (Figure 2A, Table 4).

**Table 4:** Changes in the spine, spinal cord, and femoral nerve length in volunteer dancers (*n* = 3, mean ± SD).

Imaging exam	Posture	Parameter	C1–L5	C1–7 (α)	T1–10 (β)	T11–L5 (γ)	Femoral nerves
X-ray (lateral)	Full backbend/supine	Angle (°)	151 ± 3.6/9.3 ± 2.1	44.3 ± 1.5/8.5 ± 1.5	44 ± 3.6/156.2 ± 1.0	70.3 ± 2.5/21.8 ± 6.0	232.5 ± 3.6/185.9 ± 2.3^a^
		Length (cm)	69.1 ± 1.3/54.8 ± 1.2	12.6 ± 0.8/11.4 ± 0.5	30.4 ± 3.1/20.5 ± 2.2	26.0 ± 1.7/22.9 ± 1.3	69.4 ± 8.9/61.9 ± 6.0
		Percentage (%)	126 ± 0.8	110.5 ± 2.0	148.8 ± 3.6	113.6 ± 3.2	111.7 ± 4.0
MRI (sagittal)	Partial backbend/supine	Angle (°)	56.0 ± 12.4/6.8 ± 2.6	35.1 ± 7.8/7.6 ± 0.8	162.9 ± 13.0/158.5 ± 1.6	37.6 ± 3.7/21.7 ± 7.0	
		Length (cm)	55.3 ± 2.4/54.2 ± 2.0	11.2 ± 0.9/10.8 ± 0.7	21.4 ± 1.4/21.1 ± 1.1	22.6 ± 0.7/22.3 ± 0.7	
		Percentage (%)	101.9 ± 0.7	103.5 ± 2.3	101.6 ± 1.0	101.4 ± 0.2	

^a^ Indicate the angle of the hip joint in the sagittal plane.

### Femoral nerve traction injury in an animal model

When the rats were kept in a passive full backbend posture, in which the length of the spine and femoral nerve increased to 108.7% (12.9 ± 0.2 cm/11.8 ± 0.1 cm) and 103.2% (8.7 ± 0.1 cm/8.5 ± 0.2 cm, *n* = 6) respectively (Figures 2D,F, Table 5), no symptoms of a spinal cord or nerve injury were observed. The rapid stretch with extra force on a unilateral lower extremity, in which the femoral nerve increased by 8.3% (9.4 ± 0.1 cm/8.7 ± 0.1 cm) furtherly, caused neurological impairment in both lower limbs [Basso, Beattie, and Bresnahan (BBB) locomotor scale score (12) at 4 h post-injury: left limb/right limb = 0/0, *n* = 4/30, the real-time video recording 2, see additional information 2]. The impairment manifested as weakening muscles or flaccid paralysis and made a full recovery ≤72 h.

**Table 5:** Changes in the spine and femoral nerve length of rats (*n* = 6, mean ± SD).

Imaging exam	Posture	Parameter	C1–L6	C1–7 (α)	T1–10 (β)	T11–L6 (γ)	Femoral nerves
X-ray (lateral)	Full backbend/supine	Angle (°)	123.7 ± 4.8/9.0 ± 9.1	15.3 ± 2.6/4.9 ± 3.6	45.6 ± 5.6/10.2 ± 2.4	65.4 ± 3.0/8.4 ± 3.5	207.5 ± 2.0/157.7 ± 3.7
		Length (cm)	12.9 ± 0.2/11.8 ± 0.1	2.3 ± 0.1/1.9 ± 0.2	4.3 ± 0.1/3.8 ± 0.1	6.3 ± 0.1/6.2 ± 0.1	8.7 ± 0.1/8.5 ± 0.2
		Percentage (%)	108.7 ± 1.7	121.5 ± 7.3	114.2 ± 3.2	101.7 ± 2.8	103.2 ± 1.3
	With limb pulled^a^/full backbend	Length (cm)					9.4 ± 0.1/8.7 ± 0.1
		Percentage (%)					108.3 ± 2.4

^a^ “With limb pulled” means the posture of the full backbend with the application of 25N tension to the unilateral lower limb.

No obvious abnormalities were found in subsequent morphological and pathological examination of these spinal cord specimens (Hematoxylin-eosin staining).

## Discussion

This study describes a particular type of pediatric SCIWORA that occurs during backbend practice. No apparent traumatic etiology was present before severe SCI implying that the mechanism may differ from the traumatic SCIWORA that occurs following violent events such as traffic accidents and high falls (8). It is generally believed that children's spines have good flexibility and are not prone to direct damage (13), but instead undergo transient dislocation and longitudinal stretch when force is applied. The suggested mechanism for SCIWORA caused by both violent events and backbend practices is that excessive stretching of the spine and spinal cord causes longitudinal spinal distraction injury and/or transient spinal dislocation (1). Other suggested mechanisms for the SCI caused by backbend include anatomy abnormalities, such as occult vasculopathy, spinal cord malformations, and tight filum terminale (2, 5, 14).

The current study showed that the length of the whole spine increases by 26 ± 0.8% in total and 48.8 ± 3.6% in the thoracic segment when volunteer dancers move from the lying down posture to a full backbend. We estimated that the spinal cord had the same increase. The femoral nerve length was also significantly stretched by roughly 11.7 ± 4.0%. Studies have demonstrated that while no significant structural damage occurs to the spinal column when it is stretched by 2 inches, rupture can occur when the spinal cord is pulled by only a ¼ inch (15). It is evident that excessive spinal cord longitudinal and femoral nerve distractions are the most critical anatomical factors associated with pediatric SCI caused by backbend. However, given that the children in the current study had long-term backbend training and no apparent traumatic etiology or anatomical abnormalities on the radiographic examination, it is unclear why SCI occurred. We considered potential mechanisms that may have exacerbated the neurological trauma and proposed a femoral nerve distraction injury. This may occur as a result of doing a backbend in one sudden movement or out of alignment, causing the iliopsoas and quadriceps muscles to undergo an intense eccentric contraction to keep balance, further stretching the femoral nerves and tearing the spinal cord from its attachment zone (Figure 3D). A surgical exploration of one case showed evidence of the nerve root tearing from the spinal cord. A rat model was also used to simulate the femoral nerve distraction injury to the spinal cord in a full backbend posture. Since the unilateral stretching on one lower limb resulted in impaired neurological function in both lower limbs, we concluded that mild spinal cord injury was induced in the animal model. In athletes like dancers, football players, basketball players, and long jumpers, hip hyperextension injuries are associated with acute femoral neuropathy (16), suggesting that femoral nerve traction injuries like the brachial plexus injury, are associated with trauma (17). Our mechanism for synergistic injury could explain clinical symptoms in the SCIWORA that are caused by backbends and accompanied by pain in both the back and the front of the thigh.

Results from this study also showed that the presence of tendon reflexes was associated with incomplete neurological injury and improved prognosis. This observation may help to predict the prognosis of pediatric SCIWORA caused by backbend.

One limitation is that the study was conducted using cases from one rehabilitation center and may thus not be generalizable to all populations at risk for this injury. In addition, Although rats have similar spinal cord and innervations anatomy to humans (18) and they have also been used to study nerve root traction injuries (19), a primate model should be more suitable than the rat model, and more pathological and electrophysiological evidence is needed to support our suggestion.

## Conclusion

Excessive stretching of the spinal cord and femoral nerve appears to play an important role in pediatric SCIWORA caused by backbend. A sudden eccentric contraction of the iliopsoas and quadriceps muscles used to keep balance when a backbend is out of control may be a significant aggravating factor that further stretches the femoral nerves. These findings suggest that backbend practice is dangerous for children and recommends that before doing a backbend action, adequate muscle relaxation and adaptation training is used to reduce the occurrence of pediatric SCIWORA.

## Data availability statement

The original contributions presented in the study are included in the article/**Supplementary Material**, further inquiries can be directed to the corresponding author.

## Ethics statement

The studies involving humans were approved by The China Rehabilitation Research Center Ethics Committee. The studies were conducted in accordance with the local legislation and institutional requirements. Written informed consent for participation in this study was provided by the participants’ legal guardians/next of kin. The animal study was approved by The Experimental Animal Centre of the Capital Medical University of China. The study was conducted in accordance with the local legislation and institutional requirements. Written informed consent was obtained from the minor(s)' legal guardian/next of kin for the publication of any potentially identifiable images or data included in this article.

## Author contributions

SG: Data curation, Formal Analysis, Investigation, Validation, Visualization, Writing – review & editing. HG: Data curation, Investigation, Writing – review & editing. PX: Data curation, Writing – review & editing. YX: Writing – review & editing. DY: Writing – review & editing. ZL: Writing – review & editing. YY: Writing – review & editing. LC: Writing – review & editing. YX: Writing – review & editing. MY: Writing – original draft.

## Funding

The authors declare financial support was received for the research, authorship, and/or publication of this article.

This study was supported by the Capital Funds for Health Improvement and Research under grant No. 2020-2-6012 and the China Rehabilitation Research Center Scientific Research Project under grant No. 2021zx-08.
